# Case Report: Acquired hemophilia A presenting with recurrent arthritis-like joint swelling and musculoskeletal hematomas—a focused literature review

**DOI:** 10.3389/fmed.2026.1964024

**Published:** 2026-09-30

**Authors:** Jing Zhang, Lina Zhang, Sheng-Guang Li, Ji Li, Yadan Zou, Ting Long, Ruohan Yu, Yanfeng Zhang

**Affiliations:** Department of Rheumatology and Immunology, Peking University International Hospital, Beijing, China

**Keywords:** hemophilia A, acquired, autoantibodies, hematoma, rituximab, case reports

## Abstract

**Background:**

Acquired hemophilia A (AHA) usually causes cutaneous, soft-tissue, or muscle bleeding; joint-dominant presentations are uncommon and may be mistaken for septic or inflammatory arthritis.

**Case presentation:**

A man in his late 60s with no personal or family bleeding history developed recurrent arthritis-like joint swelling, extensive bruising, and suspected iliopsoas, upper-arm, and thigh hematomas after an elective operation. For approximately 4 months, fever, raised inflammatory markers, and periarticular imaging abnormalities led to repeated infection-directed treatment. At specialist assessment, the activated partial thromboplastin time was 77.6 s. A mixing study showed greater prolongation after incubation, factor VIII activity was below 0.8%, and the factor VIII inhibitor titer exceeded 120 Bethesda units, establishing AHA. Intravenous methylprednisolone followed by a prednisone taper and two rituximab infusions was followed by cessation of new bleeding and improvement in joint symptoms. At approximately 5 months after starting immunosuppression, the inhibitor was undetectable, factor VIII activity had recovered to 27%, and the activated partial thromboplastin time was within the reporting laboratory's reference range. At telephone follow-up approximately 13 months after treatment began, he reported clinical stability for about 6 months after stopping all medications, apart from occasional brief right-knee pain; contemporaneous coagulation results were unavailable. Repeated ceftriaxone exposure was considered a possible associated factor, but causality was not established because an activated partial thromboplastin time of 40 s was documented before the first recorded postoperative exposure and later antibiotic exposures were incompletely documented.

**Conclusion:**

In an older adult, recurrent hot or swollen joints accompanied by deep-muscle bleeding and an unexplained prolonged activated partial thromboplastin time should prompt immediate factor VIII activity and inhibitor testing. This case documents inhibitor clearance with partial factor VIII recovery and subsequent patient-reported stability off treatment; it does not prove drug causality or the comparative efficacy of rituximab.

## Introduction

Acquired hemophilia A (AHA) is caused by autoantibodies that reduce factor VIII (FVIII) activity in people without congenital hemophilia. Its estimated incidence is approximately 1–1.5 cases per million persons per year and rises substantially with age. AHA often presents with spontaneous cutaneous, soft-tissue, mucosal, or muscle bleeding and a newly prolonged activated partial thromboplastin time (APTT); hemarthrosis is much less typical than in congenital hemophilia (1, 2). Delayed recognition exposes patients to ongoing bleeding and potentially hazardous invasive procedures (1, 3). Management combines prompt control of clinically relevant bleeding with inhibitor-eradication therapy and longitudinal clinical and laboratory monitoring (1, 3).

We describe AHA in a man in his late 60s whose recurrent joint swelling and musculoskeletal bleeding were repeatedly interpreted as infection or inflammatory disease. The report emphasizes the diagnostic value—and limitations—of mixing studies, the uncertainty surrounding a temporally associated ceftriaxone exposure, and the interpretation of response after glucocorticoid and rituximab treatment. A focused narrative review places the joint-dominant presentation and contemporary management in context.

## Case description

### Patient information and initial presentation

A man in his late 60s had no personal or family history of abnormal bleeding. Remote orthopedic procedures and varicose-vein surgery had not been complicated by hemorrhage. He reported no history of hypertension or diabetes. The available records did not document a pre-existing autoimmune disease or anticoagulant or antiplatelet use. Before elective inguinal hernia repair (index day 0), his hemoglobin was 133 g/L, platelet count was 238 × 10^9^/L, prothrombin time was 10.6 s, international normalized ratio was 0.96, and APTT was 40 s. The local APTT reference interval was not preserved in the transferred records, and FVIII activity was not measured. He received ceftriaxone for 3 days after surgery.

On index day 3, he fell directly onto one knee while mobilizing and developed acute swelling, inability to bear weight, and fever to 38 °C. On approximately day 11, APTT was 59.5 s and fibrinogen was 5.73 g/L. Presumed infection prompted another ceftriaxone course, without clinical improvement. Around day 13, APTT had increased to 76.8 s, C-reactive protein was 38 mg/L, and erythrocyte sedimentation rate was 46 mm/h. Pelvic and thigh magnetic resonance imaging showed extensive abnormalities in the left iliopsoas, iliacus, and peri-hip soft tissues, later considered compatible with hemorrhage. Piperacillin–tazobactam was administered for presumed deep infection. Inflammatory markers improved, but no definite infectious source was documented in the available records. During the same admission, painful swelling of the opposite upper arm and axilla developed; imaging suggested extensive soft-tissue injury or hematoma.

Over the next 3 months, minor trauma was followed by recurrent swelling of the knees, ankle, wrist, and thigh. A later 10-day cephalosporin course was recorded, but the specific agent was not documented. At a tertiary hospital, hemoglobin was 93 g/L, C-reactive protein was 128.1 mg/L, procalcitonin was 0.1 ng/mL, and APTT was 78.5 s. Thigh imaging again showed diffuse soft-tissue abnormalities. Piperacillin–tazobactam and a nonsteroidal anti-inflammatory drug were given, with partial improvement in inflammatory markers but continued episodic joint symptoms. Gum bleeding subsequently appeared. Approximately 4.5 months after the index operation, he was referred to rheumatology and immunology. The clinical timeline is summarized in Table 1.

**Table 1:** De-identified clinical timeline.

Relative time	Clinical events	Key findings	Management/outcome
Index day 0	Elective inguinal hernia repair; no prior bleeding history	APTT 40 s; PT 10.6 s; INR 0.96	Ceftriaxone for 3 days
Day 3	Fall onto knee; acute swelling, impaired weight bearing, fever	No joint-fluid confirmation	Local assessment
∼Day 11	Persistent knee symptoms	APTT 59.5 s	Ceftriaxone repeated; no improvement
∼Day 13 onward	Pelvic/thigh abnormalities; later upper-arm swelling	APTT 76.8 s; imaging compatible with possible hematomas	Piperacillin–tazobactam for presumed infection
∼Months 2–3	Recurrent joint and thigh swelling after minor trauma	Hb 93 g/L; CRP 128.1 mg/L; APTT 78.5 s	Additional antibiotic/NSAID treatment
∼Month 4	Migratory knee, ankle, and wrist swelling; gum bleeding	Persistent coagulation abnormality	Specialist referral
∼Month 4.5	AHA diagnosed	APTT 77.6 s; FVIII <0.8%; inhibitor >120 BU; time-dependent mixing pattern	Methylprednisolone 40 mg/day for 10 days, then prednisone taper
∼2 weeks after diagnosis	First rituximab infusion	No new bleeding during documented 5-month follow-up	Rituximab 600 mg
∼6 weeks after diagnosis	Second rituximab infusion	APTT improving	Rituximab 500 mg
∼5 months after IST	No new bleeding; joint symptoms improved	Inhibitor 0 BU; FVIII 27%; APTT within reporting laboratory range	Prednisone taper ongoing at 7.5 mg/day
∼7 months after IST	Medication withdrawal, reported retrospectively	CBC, urinalysis, liver/renal function, ESR and CRP reportedly normal before withdrawal; original reports unavailable	All medications stopped according to the patient
∼13 months after IST	Telephone follow-up	Clinically stable for ∼6 months off medication; occasional mild, brief right-knee pain relieved by rest	Patient-reported outcome; no contemporaneous APTT, FVIII or inhibitor results available

APTT, activated partial thromboplastin time; BU, Bethesda units; CBC, complete blood count; CRP, C-reactive protein; ESR, erythrocyte sedimentation rate; FVIII, factor VIII; INR, international normalized ratio; IST, immunosuppressive therapy; NSAID, nonsteroidal anti-inflammatory drug; PT, prothrombin time. Exact calendar dates were converted to relative time to reduce re-identification risk. No diagnostic joint aspiration was documented.

### Diagnostic assessment and differential diagnosis

At referral, examination showed marked knee swelling with restricted movement, wrist swelling, and extensive ecchymoses. APTT was 77.6 s. In a 1:1 mixing study, the patient's APTT of 76.5 s shortened to 45.6 s immediately after mixing with normal plasma but prolonged to 70.1 s after 2 h at 37 °C, supporting a time- and temperature-dependent inhibitor. FVIII activity was below 0.8%, the FVIII inhibitor titer was above 120 Bethesda units (a separately tabulated result was 121.6 BU), and an FVIII antibody assay was positive. These findings established AHA. Coagulation-factor gene exon sequencing was reported as showing no variant, but the genes tested and the indication were not specified in the available summary. Genetic testing was not required to establish AHA given the acquired bleeding phenotype and specific FVIII inhibitor findings (1, 3).

Antinuclear, antineutrophil cytoplasmic, rheumatoid factor, and anti-cyclic citrullinated peptide antibodies were negative. Immunoglobulin concentrations were reported as normal. Prostate- and liver-related tumor-marker tests were reported as negative, but the individual analytes and a systematic malignancy-screening assessment were not documented. No associated disorder was identified in the available records; this does not establish exclusion of malignancy. Ultrasonography showed knee effusions and synovial proliferation and synovial changes at the wrist and shoulder. Knee magnetic resonance imaging showed effusion, synovitis, bone-marrow edema, and chronic degenerative changes. Neither diagnostic joint aspiration nor synovial-fluid laboratory or culture results were available; the recurrent joint episodes are therefore described as arthritis-like swelling with suspected hemarthroses rather than proven hemarthrosis. Trauma could explain individual episodes but not the spontaneous upper-arm lesion, gum bleeding, or persistent APTT prolongation. The FVIII findings established AHA as the bleeding disorder but did not exclude concomitant infection or inflammatory arthritis. No specific investigations for reactive arthritis, Lyme disease, or disseminated gonococcal infection were documented. Negative autoimmune serology provided no support for lupus but did not exclude every autoimmune disorder. No von Willebrand factor antigen or activity results were available to formally assess acquired von Willebrand syndrome; the high-titer FVIII inhibitor nevertheless supported AHA.

### Therapeutic intervention, follow-up, and outcomes

The available records did not establish whether recombinant activated factor VII, activated prothrombin complex concentrate, recombinant porcine FVIII, emicizumab, or another specific hemostatic agent was administered. A contemporaneous assessment of active bleeding and any rationale for withholding hemostatic therapy were also unavailable; absence of documentation should not be interpreted as confirmed non-use. Inhibitor-eradication therapy consisted of intravenous methylprednisolone 40 mg daily for 10 days, followed by an oral prednisone taper. Rituximab 600 mg was administered approximately 2 weeks after diagnosis and 500 mg approximately 4 weeks later. Body weight, height, body-surface area, the intended rituximab protocol, and the reason for the unequal doses were not documented.

During the first approximately 5 months after treatment began, no new bleeding was reported, and joint swelling and mobility improved (Figures 1, 2). Approximately 1 month after the first rituximab infusion, FVIII activity had increased to 3.2% and the inhibitor had fallen to 98 BU. At the last available coagulation assessment, approximately 5 months after starting immunosuppression, APTT was within the reporting laboratory's reference range, the inhibitor was undetectable, FVIII activity was 27%, and no new bleeding was reported while prednisone was still being tapered at 7.5 mg/day (Figure 3). These laboratory findings documented inhibitor clearance with partial FVIII recovery, not complete remission at that time.

**Figure 1:**
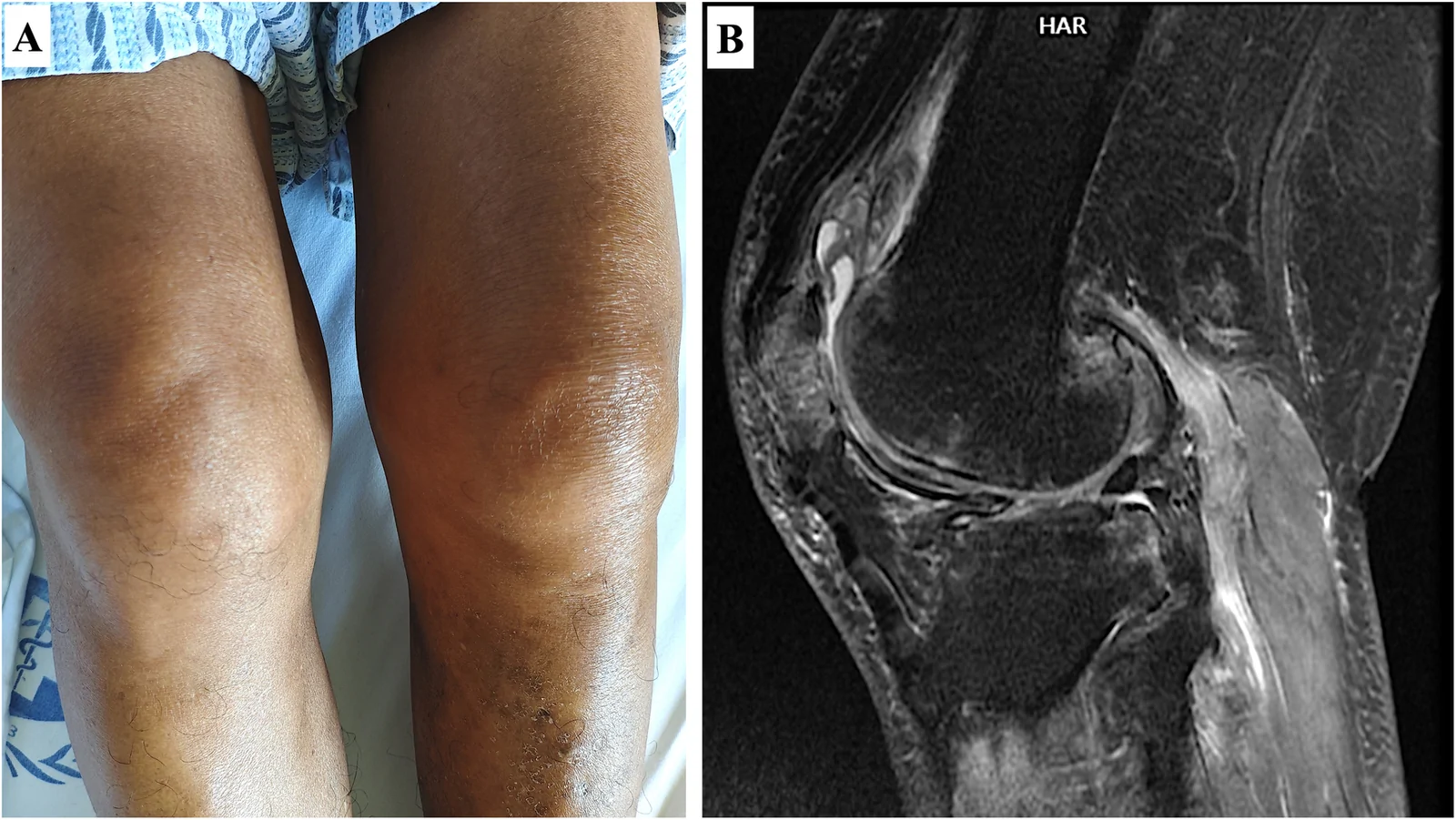
Baseline knee appearance and selected sagittal left-knee magnetic resonance image. **(A)** Marked asymmetric knee swelling with restricted extension. **(B)** The selected image shows joint effusion, synovial change, and chronic structural abnormalities; this image alone does not confirm intra-articular blood. The displayed clinical photograph and image have been cropped and exported without embedded patient metadata.

**Figure 2:**
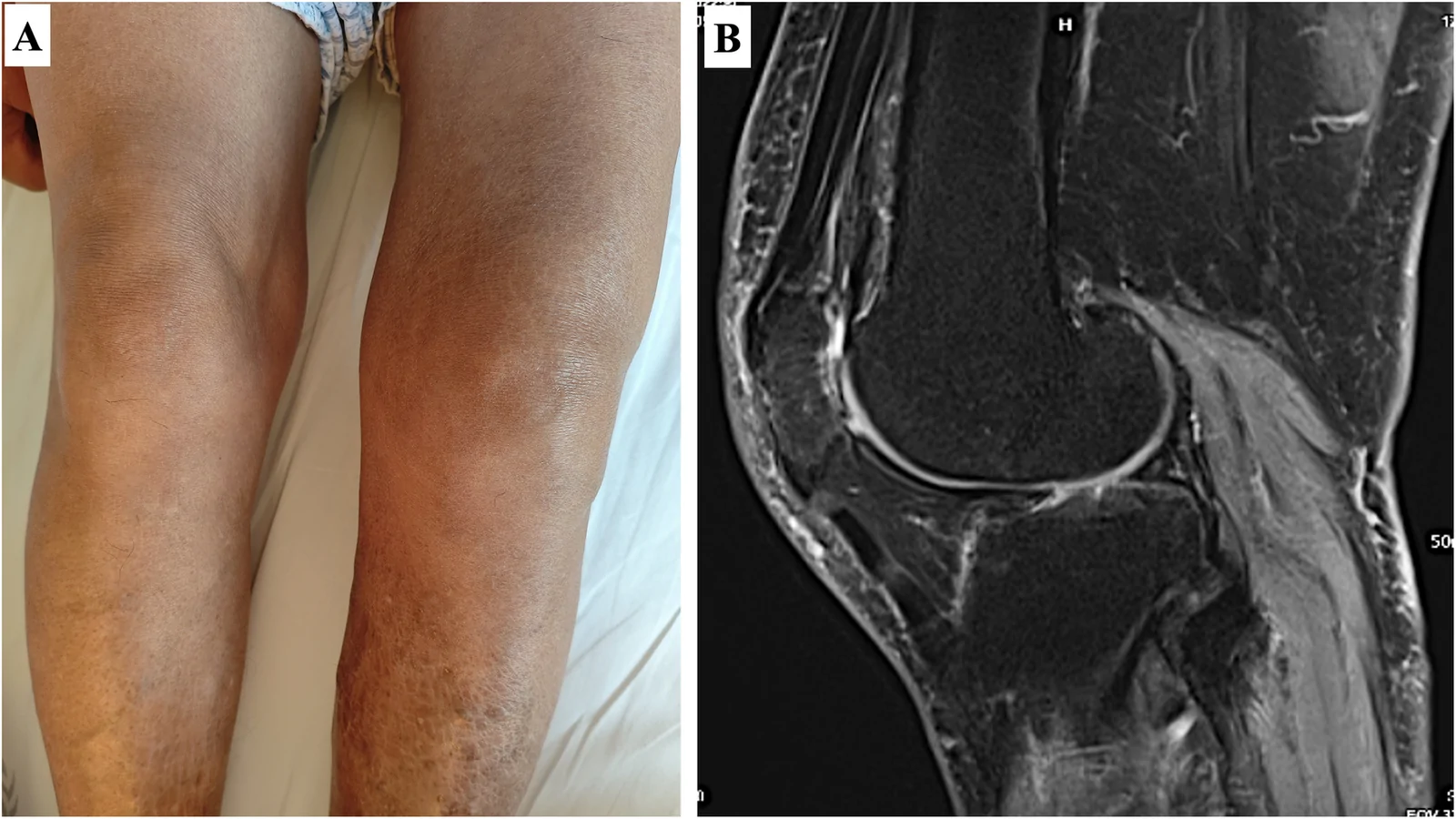
Follow-up knee appearance and selected sagittal left-knee magnetic resonance image after immunosuppression. **(A)** Reduced knee swelling and improved extension. **(B)** Reduced effusion and synovial change were observed, while chronic structural abnormalities remained. The paired images illustrate evolution and do not establish complete radiographic resolution.

**Figure 3:**
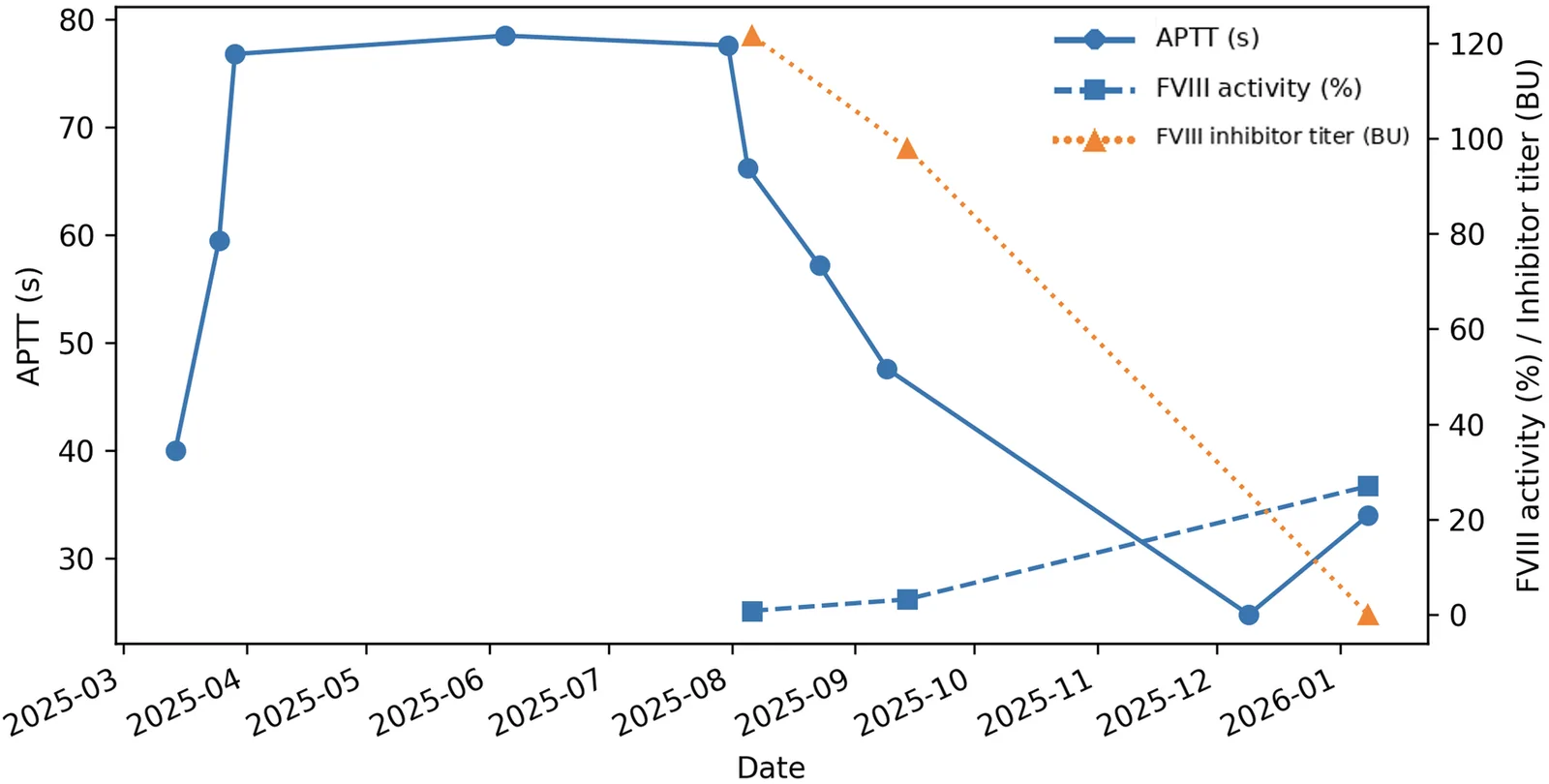
Serial laboratory measurements during the clinical course. The horizontal axis includes both the prediagnostic period and approximately 5 months of follow-up after immunosuppression began; it does not represent 11 months of post-treatment observation. Solid blue circles show activated partial thromboplastin time (APTT) on the left axis; dashed blue squares show factor VIII (FVIII) activity and dotted orange triangles show FVIII inhibitor titer on the right axis. Only observed measurements are plotted; connecting lines aid visualization and do not imply values between measurements. Medication withdrawal and the later telephone follow-up are summarized in Table 1 but are not plotted because no corresponding coagulation results were available.

At telephone follow-up in September 2026, approximately 13 months after immunosuppression began, the patient reported that he had stopped all medications in March 2026 and had remained clinically stable for about 6 months. He described occasional mild, brief right-knee pain that resolved with rest. He also reported normal complete blood count, urinalysis, liver and renal function tests, erythrocyte sedimentation rate, and C-reactive protein before medication withdrawal. The original reports from those tests were not available for review. No contemporaneous APTT, FVIII activity, or inhibitor results were available at the telephone follow-up.

## Discussion and focused review of the literature

### Joint-dominant presentation and diagnostic delay

This case illustrates a rheumatologic diagnostic trap: recurrent hot or swollen joints, fever, raised inflammatory markers, and periarticular imaging abnormalities can draw attention toward septic or inflammatory arthritis while deep-muscle and soft-tissue bleeding is evolving. A focused PubMed search was updated on 9 August 2026 using combinations of “acquired hemophilia A/haemophilia A” and “hemarthrosis,” “arthritis,” or “joint”; this was a narrative, not systematic, review. Published joint-dominant reports remain uncommon. Wendling and Bertrand described two patients with recurrent hemarthroses (4). Later reports included shoulder hemarthrosis with immune thrombocytopenia (5), an elbow presentation initially considered septic arthritis or a synovial tumor and complicated by compartment syndrome (6), and an older adult with hemarthrosis accompanied by mucosal and cutaneous bleeding (7). Unlike those reports, the present patient had migratory arthritis-like swelling plus suspected iliopsoas, upper-arm, and thigh hematomas over several months. The absence of diagnostic arthrocentesis is important: intra-articular bleeding is clinically plausible but not directly proven.

The earliest durable clue was the progressive APTT prolongation. An incubated mixing study can support a time-dependent inhibitor, but mixing tests are poorly standardized and cannot independently establish or exclude AHA or reliably distinguish an FVIII inhibitor from lupus anticoagulant; both may coexist. Specific FVIII activity and a validated inhibitor assay are therefore decisive, with chromogenic FVIII testing or lupus-anticoagulant-directed studies used when interference is suspected (1, 3, 8). For rheumatologists, surgeons, and infection specialists, the practical trigger is unexplained bleeding or hematoma plus a newly or persistently prolonged APTT—not the severity of joint findings alone.

### Causality and associated conditions

Approximately half of AHA cases have no identified associated condition (1, 2). Drug-associated AHA has been described, but historical systematic reviews and contemporary pharmacovigilance analyses are dominated by spontaneous reports, heterogeneous drugs, and incomplete information about alternative causes (9, 10). The index record documented an APTT of 40 s before the first postoperative ceftriaxone exposure; because the local reference interval was unavailable, this value neither proves nor excludes an evolving inhibitor. Later cephalosporin exposure was incompletely specified, additional beta-lactams were given, and the coagulopathy persisted after drug withdrawal. Surgery itself has also preceded AHA in published cases (11). Accordingly, ceftriaxone is best recorded as a possible associated exposure, not an established cause. Idiopathic AHA or another unrecognized trigger remains possible, and this case does not justify avoidance of all beta-lactam antibiotics.

### Hemostatic and inhibitor-eradication treatment

Management has two distinct aims: rapid control of clinically relevant bleeding and eradication of the inhibitor. Current recommendations favor recombinant activated factor VII, activated prothrombin complex concentrate, or recombinant porcine FVIII for clinically relevant bleeding irrespective of FVIII activity or inhibitor titer (1, 3, 12). The incomplete hemostatic-treatment documentation in this case does not support a decision to withhold such therapy. Persistent, severe focal hemorrhage despite initial pharmacologic hemostatic therapy may occasionally require multidisciplinary rescue intervention. A recent report described selective endovascular embolization for an expanding quadriceps hematoma with arterial extravasation and hemodynamic instability despite initial recombinant activated factor VII, with intensified factor therapy continued as procedural cover (13).

FVIII activity below 1 IU/dL and an inhibitor titer above 20 BU are adverse prognostic features for inhibitor eradication and support combined immunosuppression in appropriately selected patients (1, 14). Registry data suggested higher stable complete-remission rates with glucocorticoid plus cyclophosphamide than with glucocorticoid alone, while rituximab-based regimens were heterogeneous and subject to confounding by indication (15). Two 2024 randomized trials have strengthened the comparative evidence: one found single-dose rituximab plus glucocorticoid noninferior to cyclophosphamide plus glucocorticoid, whereas another found similar overall complete-remission rates but clinically relevant subgroup differences (16, 17). Thus, the present inhibitor clearance shows that a rituximab-containing regimen was feasible in this patient; a single case receiving concomitant glucocorticoids cannot establish rituximab's independent effect, optimal dose, or comparative safety.

Emicizumab provides a non-factor strategy for bleeding prophylaxis but does not eradicate the FVIII inhibitor. Prospective single-arm phase 2 and phase 3 studies support its prophylactic activity in selected patients, and expert recommendations emphasize its interference with APTT-based assays and the need for specialized laboratory coordination (18–20). Its use was not documented in this case.

### Outcome, strengths, and limitations

The disappearance of the inhibitor, absence of new bleeding, and recovery of FVIII to 27% at approximately 5 months represented a meaningful response but did not meet definitions of complete remission requiring normal FVIII activity and withdrawal of immunosuppression. Relapse remains possible; serial FVIII activity and inhibitor measurements are more informative than APTT alone during follow-up (1, 3). Coordinated hematology and rheumatology care, orthopedic input when needed, and cautious physical therapy after bleeding control may support functional recovery. Active bleeding requires clinical reassessment, serial blood counts, and site-specific examination or imaging (1). During inhibitor-eradication therapy, FVIII activity and inhibitor titer should be assessed approximately weekly until a response is observed or a change in immunosuppression is planned, with surveillance for treatment toxicity (3). After complete remission, international recommendations advise FVIII monitoring monthly for 6 months, every 2–3 months until 12 months, and every 6 months during the second year and beyond if possible (1). The later telephone report supports sustained patient-reported clinical improvement off treatment. However, the reported normal routine laboratory tests cannot establish FVIII normalization or inhibitor eradication; complete remission cannot be confirmed without updated coagulation results. The intermittent knee pain cannot be attributed to recurrent hemarthrosis or another cause without examination and appropriate testing.

Strengths include a longitudinal clinical and laboratory record, specific inhibitor testing, and paired clinical images. Limitations include the lack of joint-fluid confirmation of hemarthrosis, incomplete external laboratory reference intervals, no documented lupus anticoagulant-directed testing, incomplete antibiotic exposure data, incomplete hemostatic-treatment and etiologic-investigation records, coagulation measurements limited to approximately 5 months after treatment began and later follow-up based on patient report without contemporaneous coagulation results, and inability to separate the effects of glucocorticoids from rituximab. These limitations constrain conclusions about joint bleeding, drug causality, and treatment efficacy but do not weaken the laboratory diagnosis of AHA.

## Conclusion

AHA should be considered when recurrent arthritis-like joint swelling occurs with musculoskeletal hematomas, mucosal bleeding, or an unexplained prolonged APTT. Prompt FVIII activity and inhibitor testing can prevent months of diagnostic delay. In this patient, combined glucocorticoid and rituximab treatment was followed by cessation of bleeding, inhibitor clearance, and partial FVIII recovery, followed by patient-reported clinical stability off medication. The clinical course supports careful recognition and longitudinal monitoring, not a causal claim about ceftriaxone or a comparative efficacy claim for rituximab.

## Patient perspective

The patient reported marked relief after treatment because the bleeding and joint symptoms improved. After several months without a clear explanation for his illness, he expressed gratitude that the diagnosis had finally been established and that treatment had helped bring him out of a prolonged period of uncertainty. During the later telephone follow-up, he described himself as fully recovered after treatment at our hospital, although he still experienced occasional mild, transient right-knee pain.

## Data Availability

The original contributions presented in the study are included in the article/Supplementary Material, further inquiries can be directed to the corresponding authors.
